# Quantitative proteomics identifies a plasma multi-protein model for detection of hepatocellular carcinoma

**DOI:** 10.1038/s41598-020-72510-9

**Published:** 2020-09-23

**Authors:** Zhenhua Du, Xinyi Liu, Xiaojun Wei, Hongbo Luo, Peiyao Li, Mengting Shi, Bingqian Guo, Ying Cui, Zhenglin Su, Jifeng Zeng, Anfeng Si, Pengbo Cao, Gangqiao Zhou

**Affiliations:** 1 State Key Laboratory of Proteomics, National Center for Protein Sciences at Beijing, Beijing Proteome Research Center, Beijing Institute of Radiation Medicine, 27 Taiping Road, Beijing, 100850 People’s Republic of China; 2 Medical College of Guizhou University, Guiyang City, 550025 People’s Republic of China; 3 Affiliated Cancer Hospital of Guangxi Medical University, Nanning City, 530021 People’s Republic of China; 4 The No. 954 Hospital of PLA, Shannan City, 856100 People’s Republic of China; 5 Department of Surgical Oncology, Jinling Hospital, School of Medicine, Nanjing University, 305 Zhongshan East Road, Nanjing City, 210002 People’s Republic of China; 6 Collaborative Innovation Center for Personalized Cancer Medicine, Center for Global Health, School of Public Health, Nanjing Medical University, Nanjing City, 211166 People’s Republic of China

**Keywords:** Hepatocellular carcinoma, Diagnostic markers

## Abstract

More efficient biomarkers are needed to facilitate the early detection of hepatocellular carcinoma (HCC). We aimed to identify candidate biomarkers for HCC detection by proteomic analysis. First, we performed a global proteomic analysis of 10 paired HCC and non-tumor tissues. Then, we validated the top-ranked proteins by targeted proteomic analyses in another tissue cohort. At last, we used enzyme-linked immunosorbent assays to validate the candidate biomarkers in multiple serum cohorts including HCC cases (HCCs), cirrhosis cases (LCs), and normal controls (NCs). We identified and validated 33 up-regulated proteins in HCC tissues. Among them, eight secretory or membrane proteins were further evaluated in serum, revealing that aldo–keto reductase family 1 member B10 (AKR1B10) and cathepsin A (CTSA) can distinguish HCCs from LCs and NCs. The area under the curves (AUCs) were 0.891 and 0.894 for AKR1B10 and CTSA, respectively, greater than that of alpha-fetoprotein (AFP; 0.831). Notably, combining the three proteins reached an AUC of 0.969, which outperformed AFP alone (*P* < 0.05). Furthermore, the serum AKR1B10 levels dramatically decreased after surgery. AKR1B10 and CTSA are potential serum biomarkers for HCC detection. The combination of AKR1B10, CTSA, and AFP may improve the HCC diagnostic efficacy.

## Introduction

Hepatocellular carcinoma (HCC) is one of the highly lethal malignancies and accounts for 75–85% of primary liver cancers^1^. About 841 080 new cases and 781 631 deaths occurred worldwide during 2018, among which more than 50% were in China^1^. Risk factors for HCC include hepatitis B/C viral infections, obesity, diabetes, aflatoxin, alcohol, and nonalcoholic fatty liver^2^. HCC patients diagnosed at early stages have good chances to receive beneficial treatments, and the 5-year survival rate can reach 50–75%^3^. However, more than 60% of patients are diagnosed at advanced stages with a 5-year survival rate of less than 16%^4^. Therefore, development of effective strategies to detect HCC in high-risk populations could improve treatment outcomes.

Imaging examinations and serological biomarker tests represent the major methods for HCC detection. Hepatic ultrasound (US) is now a widely used imaging method for HCC surveillance, but its performance varies by as much as 23–90% in sensitivity^5^. Other imaging methods such as computed tomography (CT) and magnetic resonance imaging (MRI) can exceed sensitivity of 80%, but tend to be reserved for patients at highest risk since they are expensive and uncomfortable^6^. Serological biomarkers are also important tools for HCC detection. To date, the clinically used HCC diagnostic biomarkers include alpha-fetoprotein (AFP), Lens culinaris agglutinin A-reactive fraction of alpha-fetoprotein (AFP-L3) and des-gamma-carboxy prothrombin (DCP), but their performance are limited. For the clinically diagnosed HCC, the sensitivity and specificity are only 41–65% and 80–90% for AFP^7^, 56–77% and 82–87% for DCP^8^, and 28–56% and 92–97% for AFP-L3^9^, respectively. Over the past decade, many potential non-protein serum markers have been reported, such as circulating tumor DNA mutations, circulating tumor DNA methylation and microRNAs^10^. However, serum protein markers are the most applicable in clinical contexts, because generally such tests are at low cost, highly reproducible, and almost free of sample pretreatments (e.g., extraction, purification or reverse transcription)^11^. Although several potential protein diagnostic biomarkers have been proposed, such as glypican 3 (GPC3)^12^, osteopontin (OPN)^13^ and golgi protein-73 (GP73)^14^, few of them have been moved into clinical practice, and the reports concerning their performance are conflicting^15, 16^. Therefore, comprehensive studies are needed to identify more efficient biomarkers for HCC diagnosis alone or in combination.

Isobaric tagging for relative and absolute quantitation (iTRAQ)-based proteomic analysis has been considered as a powerful proteome-wide technology for screening potential cancer biomarkers, as its high throughput, high stability and free of the restriction of sample property^17^. It has been proven to be useful in discovery biomarkers for HCC, such as the discovery of OPN, S100 calcium binding protein A9 (S100A9), and N-myc downstream regulated 1 (NDRG1) for HCC detection^18–20^. Besides, parallel reaction monitoring (PRM)-based mass spectrometry (MS) is widely used in targeted proteomic analyses to verify the dysregulation of candidate proteins^21^. The combination of iTRAQ-based and PRM-MS-based proteomic analyses has been applied in biomarker investigations and shown to be a more high-throughput strategy^22, 23^. For examples, Cui et al*.* investigated the serum proteomic differences between patients with early recurrent spontaneous abortion and healthy controls to identify potential biomarkers by combining iTRAQ-based discovery analyses and PRM-MS-based verification analyses^22^. However, this integrative strategy has not been employed for identifying HCC biomarkers.

In this study, we aimed to identify serum biomarkers distinguishing HCC patients from healthy and high-risk controls. For that, we first performed an iTRAQ-based global proteomic analysis in tissue samples. Then, we used PRM-MS analyses to validate the potential proteins in independent tissue samples. Further, the candidate biomarkers were validated in serum samples by enzyme-linked immunosorbent assays (ELISAs). Finally, we found that AKR1B10 and CTSA were potential serum biomarkers for HCC detection.

## Materials and methods

### Study participants

The recruited participants were defined as HCC cases (HCCs), liver cirrhosis cases (LCs) or healthy normal controls (NCs) by medical doctors, according to eligibility criteria listed in the Supplementary Table S1. HCC was diagnosed on the basis of US, CT, or MRI characteristics and was confirmed by histopathology according to the American association for the study of liver diseases (AASLD) guidelines^42^. Tumor stage was defined according to the Barcelona clinic liver cancer (BCLC) classification staging system^43^. LCs were confirmed by biopsy or two imaging technologies (hepatic ultrasound with CT or MRI). The NCs had no history of liver or other systematic diseases. US was also done to ensure that no HCC was present in LCs and NCs. The major characteristics of all participants were shown in Table 1. For each group across different cohorts, the age and sex were well matched. However, the BCLC stages differed among HCCs in the discovery cohort and validation cohorts: more patients in the discovery cohort and the tissue validation cohort have early-stage HCCs.

**Table 1:** Major characteristics of the participants.

Cohorts	Sample types	Groups	Sample numbers	Age, years [median (min–max)]	Sex (male/female)	HBsAg (positive/negative)	HCV antibody (positive/negative)	BCLC (A/B/C/–)
DSC (n = 10)	Tissue	HCC	10	53 (39–65)	9/1	10/0	0/10	7/1/2/0
TVC (n = 5)		HCC	5	53 (42–57)	5/0	5/0	0/5	5/0/0/0
SVC1 (n = 24)	Serum	NC	8	42 (37–54)	7/1	0/8	0/8	–
		LC	8	40 (35–50)	8/0	8/0	0/8	–
		HCC	8	53 (46–62)	7/1	8/0	0/8	3/2/1/2
SVC2 (n = 40)		NC	16	44 (36–55)	14/2	0/16	0/16	–
		LC	16	41 (34–51)	13/3	16/0	0/16	–
		HCC	8	53 (47–63)	6/2	8/0	0/8	2/2/3/1
FUC (n = 21)		HCC	21	59 (20–78)	17/4	21/0	0/21	5/14/2/0
*P*^a^			–	0.08	0.17	–	–	< 0.01

*BCLC* Barcelona Clinic Liver Cancer stage, *DSC* discovery cohort, *FUC* follow-up cohort, *HBsAg* hepatitis B virus surface antigen, *HCC* hepatocellular carcinoma, *HCV* hepatitis C virus, *LC* liver cirrhosis, *NC* healthy normal control, *SVC1* serum validation cohort 1, *SVC2* serum validation cohort 2, *TVC* tissue validation cohort.

– not available.

^a^*P* values for the differences among different groups from different cohorts (*χ*^2^ test).

### Ethics approval and consent to participate

The research protocol was approved by the institutional ethics committees of No 81 Hospital of PLA (Nanjing city, China), Affiliated Tumor Hospital of Guangxi Medical University (Nanning city, China), and Beijing Institute of Radiation Medicine (Beijing, China). This study was conducted in accordance with the Declaration of Helsinki. Written informed consent was obtained from each participant.

### Samples collection and processing

The liver tissues were collected at the time of surgery and immediately stored in liquid nitrogen after washing with ice-cold phosphate-buffered saline (PBS). Meanwhile, part of the tissues was formalin-embedded and used for confirming the tumor or non-tumor regions. Each specimen was reviewed by a board-certified pathologist to confirm that the frozen sections were histologically consistent with the tumor or non-tumor tissues, and the tumor sections must contain more than 70% tumor cell nuclei and less than 20% necrosis.

Blood samples were collected using the vacutainer tubes (Becton, NJ, USA) without anticoagulant and allowed to clot at room temperature for 30 min before centrifugation at 3 000 g for 10 min. Supernatants (serum) were collected, immediately aliquoted in sterile centrifuge tubes and frozen at − 80 °C until being used. Deidentified human serum samples were coded before being tested by ELISAs, and then the coded data were analyzed and interpreted by the statistic team independently.

### Proteomic analyses

In the discovery stage, quantitative mass spectrometry analyses of tissue samples were done using the iTRAQ-based liquid chromatography-mass spectrometry/mass spectrometry analysis. Notably, a total of three iTRAQ 8-plex kits were used to label the 20 tissue samples and quality controls (QC) that were prepared by mixing aliquots of each non-tumor sample and used to monitor mass spectrometry operation and correct batch effects. The eight iTRAQ tags (113, 114, 115, 116, 117, 118, 119 and 121) in the first kit were used to label seven tumor specimens and one QC; the eight iTRAQ tags in the second kit were used to label the corresponding seven non-tumor specimens and one QC; the last kit was used to label the remaining 3 paired tissue specimens and one QC. In the tissue validation stage, targeted proteomic analyses were done using PRM-MS. Notably, a total of four quantitative concatamer (QconCAT) genes that covered separately 13, 12, 12, and 13 proteins were constructed in Sangon Biotech (Shanghai, China) (Supplementary Table S2). Details on tissue sample preparation and mass spectrometry analysis are provided in the Supplementary material (available online).

### Pathway enrichment analyses

The Kyoto Encyclopedia of Genes and Genomes (KEGG) pathway enrichment analyses were performed by the online tool Metascape using default parameters. Significantly enriched pathways were determined according to the criteria: *P* < 0.05 (Banjamini–Hochberg correction), gene counts ≥ 3, and enrichment factor > 1.5. *P* values were calculated based on the accumulative hypergeometric distribution.

### Enzyme-linked immunosorbent assays (ELISAs)

Based on the PRM-MS validation results, we chose these proteins that were annotated as membrane or secretory proteins in GeneCard or human protein atlas (HPA) database as potential serum biomarkers. Then, the serum levels of these candidate biomarkers were determined using the following ELISA kits: aspartate beta-hydroxylase (ASPH) (1:2; #OKEH04669-BJ07242018, Aviva Systems Biology, CA, USA), calnexin (CANX) (1:10; #OKCD00229, Aviva Systems Biology, CA, USA), cathepsin A (CTSA) (1:5; #OKEH01165-BJ07242018, Aviva Systems Biology, CA, USA), cytoskeleton associated protein 4 (CKAP4) (1:20; #OKCA01191, Aviva Systems Biology, CA, USA), poly (ADP-ribose) polymerase 1 (PARP1) (1:2; #OKEH00674-BJ07242018, Aviva Systems Biology, CA, USA), nucleoside diphosphate kinase A (NME1) (1:5; #OKEH02350, Aviva Systems Biology, CA, USA) and AFP (1:5; #KA0202, Abnova, Taiwan, China). The assays for aldo–keto reductase family 1 member B10 (AKR1B10) and cytochrome b-245 beta chain (CYBB) were developed in-house. For all ELISA experiments, each sample was assayed in duplicate, and the absorbance was measured with a Tecan’s Sunrise microplate reader (Tecan Sunrise, Zürich, Switzerland). Additional details on ELISAs were provided in Supplementary material (available online).

### Experimental design and statistical rationale

**Figure 1:**
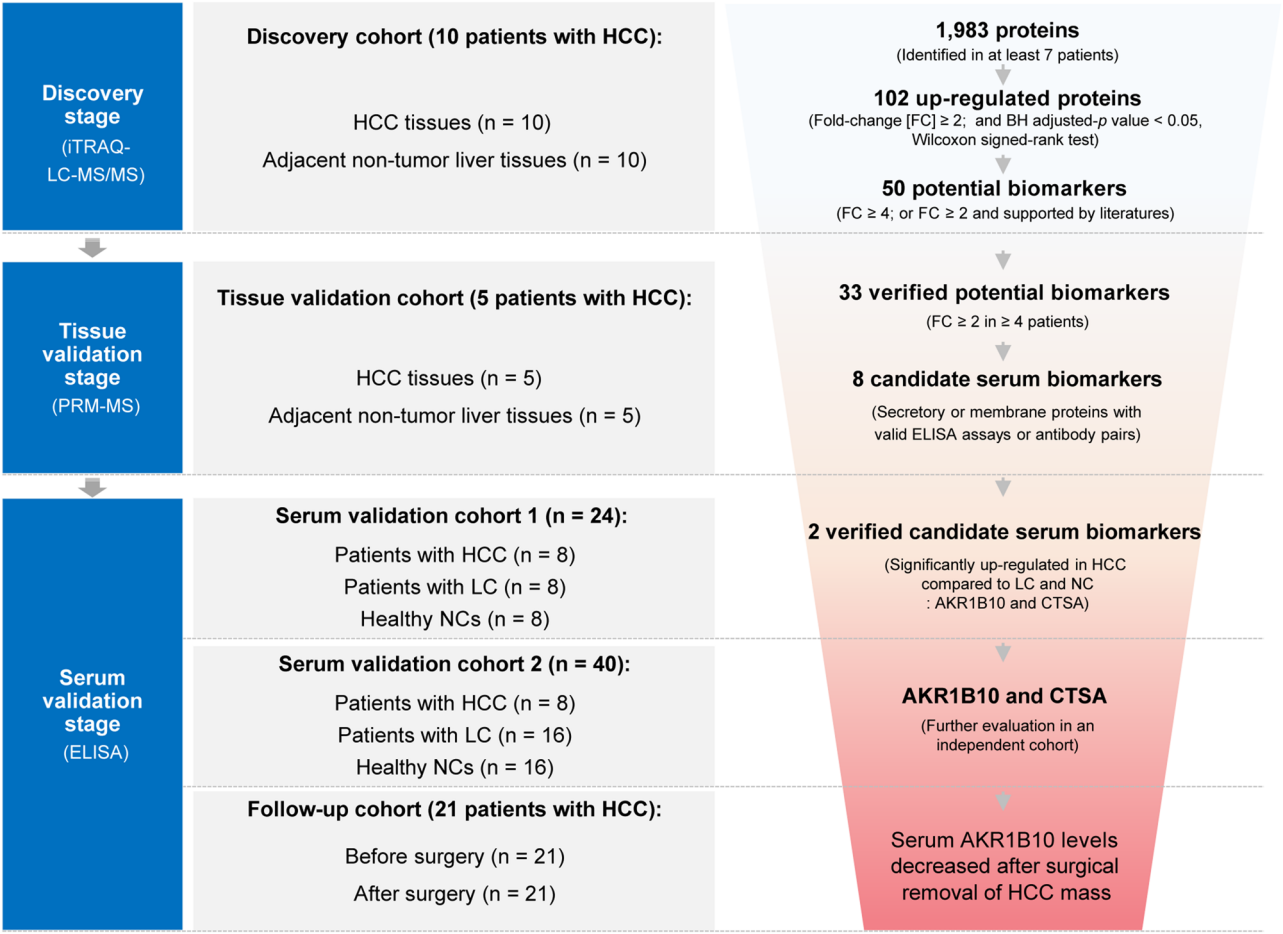
Study workflow. This study was comprised of three stages. First, iTRAQ-LC–MS/MS-based proteomic analyses were performed in the discovery cohort to screen the potential biomarkers. Then, they were validated in the tissue validation cohort by PRM-MS assays. Next, the candidate biomarkers were further sequentially validated by ELISA in the serum validation cohort 1 and 2. Finally, AKR1B10 and CTSA proteins survived. Additionally, the follow-up cohort, in which the surgical HCC patients were followed up one week, was used to evaluate the serum AKR1B10 levels change after operation. AKR1B10, aldo–keto reductase family 1 member B10; BH, Benjamin-Hochberg correction; CTSA, cathepsin A; ELISA, Enzyme-linked immunosorbent assay; HCC, hepatocellular carcinoma; iTRAQ-LC–MS/MS, isobaric tagging for relative and absolute quantification-based liquid chromatography-mass spectrometry/mass spectrometry; LC, liver cirrhosis; NC, normal control; PRM-MS, parallel reaction monitoring-based mass spectrometry.

This study was comprised of three stages including a total of 100 participants in five cohorts (Fig. 1, Table 1 and Supplementary Table S3). (1) The discovery stage contained a discovery cohort of ten paired HCC and adjacent non-tumor tissues for exploring the HCC proteomes and identifying differentially expressed proteins by iTRAQ-based proteomic analyses.(2) The tissue validation stage included a tissue validation cohort of five paired HCC and adjacent non-tumor tissues for validating the candidate up-regulated proteins by PRM-MS analyses. (3) The serum validation stage included two independent serum validation cohorts (designated as the serum validation cohort 1 and 2), consisting of 16 HCC cases (HCCs), 24 liver cirrhosis cases (LCs) and 24 healthy normal controls (NCs), for the sequential validation of candidate biomarkers by ELISAs. Furthermore, an independent follow-up cohort containing 21 HCC patients were recruited and followed up for one week after surgery to investigate the dynamic changes of the candidate biomarkers.

All statistical analyses were done with the Statistical Package for the Social Sciences (SPSS 24, SPSS Inc., NY, USA) software, MedCalc (version 10.4.7.0; Medcalc Software, Mariakerke, Belgium) and the GraphPad Prism package (version 5.0; GraphPad Software, CA, USA). Continuous variables were expressed as the median (min–max) or mean ± standard error, whereas categorical variables were expressed as numbers. Mann–Whitney test was applied to compare the circulating protein levels between groups. Receiver operating characteristic (ROC) curves were applied to evaluate sensitivity, specificity, and respective areas under the ROC curves (AUCs) with 95% confidence interval. The optimal cut-point value in ROC analysis was defined by Youden index. The combination of biomarkers (AKR1B10, CTSA and AFP) was constructed using the logistic regression. The predicted probability of being detected as HCC by the combination was calculated by the following formula: logit [*P* = HCC] = *β*_*1*_ × level of biomarker_1_ + *β*_*2*_ × level of biomarker_2_ + … *β*_*n*_ × level of biomarker_n_. *P* value (two-sided) < 0.05 was considered to be statistically significant.

## Results

### Screening of candidate biomarkers for HCC by iTRAQ-based proteomic analyses

**Figure 2:**
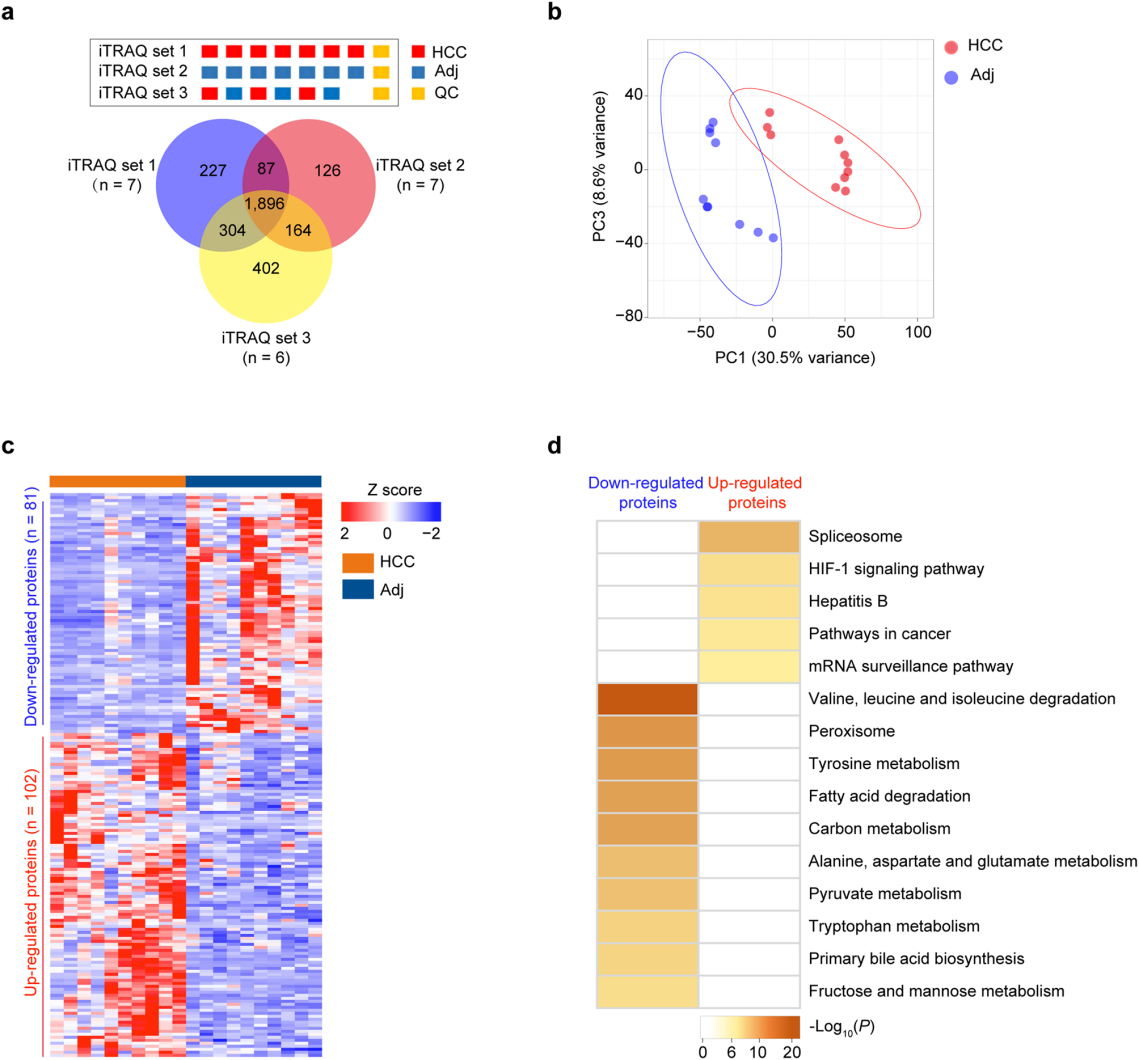
iTRAQ-based proteomic analyses of HCC tissues from the discovery cohort. **a** The iTRAQ labeling strategy (up) and the Venn plot of numbers of proteins identified by the three sets of iTRAQ-based proteomic analyses (down). Three sets of iTRAQ 8-plex kits were used to label those 20 tissue samples, and the pooled quality control samples (QCs) that were prepared by mixing aliquots of each non-tumor sample: the iTRAQ set 1 labeled 7 HCCs and 1 QC; the iTRAQ set 2 labeled 7 adjacent non-tumor samples (Adj) and 1 QC; and the iTRAQ set 3 labeled 3 HCC, 3 Adj, and 1 QC. **b** The principal component analysis (PCA) of HCC tissues and adjacent non-tumor liver tissues based on the proteomic data. **c** The expression heatmap of the 183 differentially expressed proteins (DEPs) between HCC tissues and adjacent non-tumor liver tissues. DEPs were defined as those proteins with log_2_-transformed fold-change (HCC/Adj) ≥ 1 or ≤ − 1, and Benjamin-Hochberg adjusted *P* value < 0.05 (Wilcoxon signed-rank test). **d** Kyoto Encyclopedia of Genes and Genomes (KEGG) pathways enriched by DEPs using Metascape. HCC, hepatocellular carcinoma; iTRAQ, isobaric tagging for relative and absolute quantification.

To obtain a comprehensive proteomic landscape of HCC and identify potential biomarkers, the paired tumor and adjacent non-tumor liver tissues of 10 HCC patients (designated as the discovery cohort; Table 1) were subjected to iTRAQ-based proteomic analyses. The tissues were analyzed by three sets of iTRAQ 8-plex experiments (Supplementary methods). The mass spectrometry platform was stable and repeatable as judged by QC runs during the entire data-collecting period (Supplementary Fig. S1). A total of 2 514, 2 273 and 2 766 proteins, respectively, were identified (number of unique peptides ≥ 2, FDR < 0.01; Fig. 2a). To increase the reliability, we chose the 1 983 proteins detected in at least 7 pairs of samples for further analyses. The principle component analyses (PCA) showed that the proteomic data could separate the HCC tissues from the adjacent non-tumor liver tissues (Fig. 2b), revealing an altered proteomic landscape of HCCs.

Differential expression analyses showed that a total of 183 proteins were significantly dysregulated in HCC tissues compared to the adjacent non-tumor liver tissues (*P* < 0.05, log_2_-transformed fold-change ≥ 1 or ≤ − 1; Fig. 2c), of which 102 ones were up-regulated and 81 ones were down-regulated (Supplementary Table S4). KEGG pathway enrichment analyses showed that the up-regulated proteins were significantly enriched in pathways related to cancers (Fig. 2d and Supplementary Fig. S2), including spliceosome, HIF-1 pathway, Hepatitis B, pathways in cancer, and mRNA surveillance pathway. Notably, spliceosome is the most activated pathway, with the components of small nuclear ribonucleoprotein polypeptides (SNRPB2, SNRPD1, and SNRPD2), heterogeneous nuclear ribonucleoprotein proteins (HNRNPC and HNRNPK), serine and arginine rich splicing factor 3 (SRSF3), RNA binding motif protein 8A (RBM8A) and cell division cycle 5 like protein (CDC5L) over-expressed in HCC tissues (Supplementary Fig. S3). Consistently, the activation of spliceosome pathway has also been shown in other studies. For examples, a meta-analysis of publicly available gene expression datasets revealed that the spliceosome pathway is activated in HCC^24^. Another study also showed that the spliceosome pathway is over-represented in the up-regulated proteins of early-stage HCC tissues^25^. Together, these results suggested the spliceosome pathway might play important roles in HCC tumorigenesis. In contrast, the down-regulated proteins were significantly enriched in metabolism pathways (Fig. 2d), including valine, leucine, and isoleucine degradation, peroxisome and tyrosine metabolism. This finding was consist with the previous studies^25, 26^, suggesting that the deficiency of normal liver metabolism function might be a common feature of HCC.

**Figure 3:**
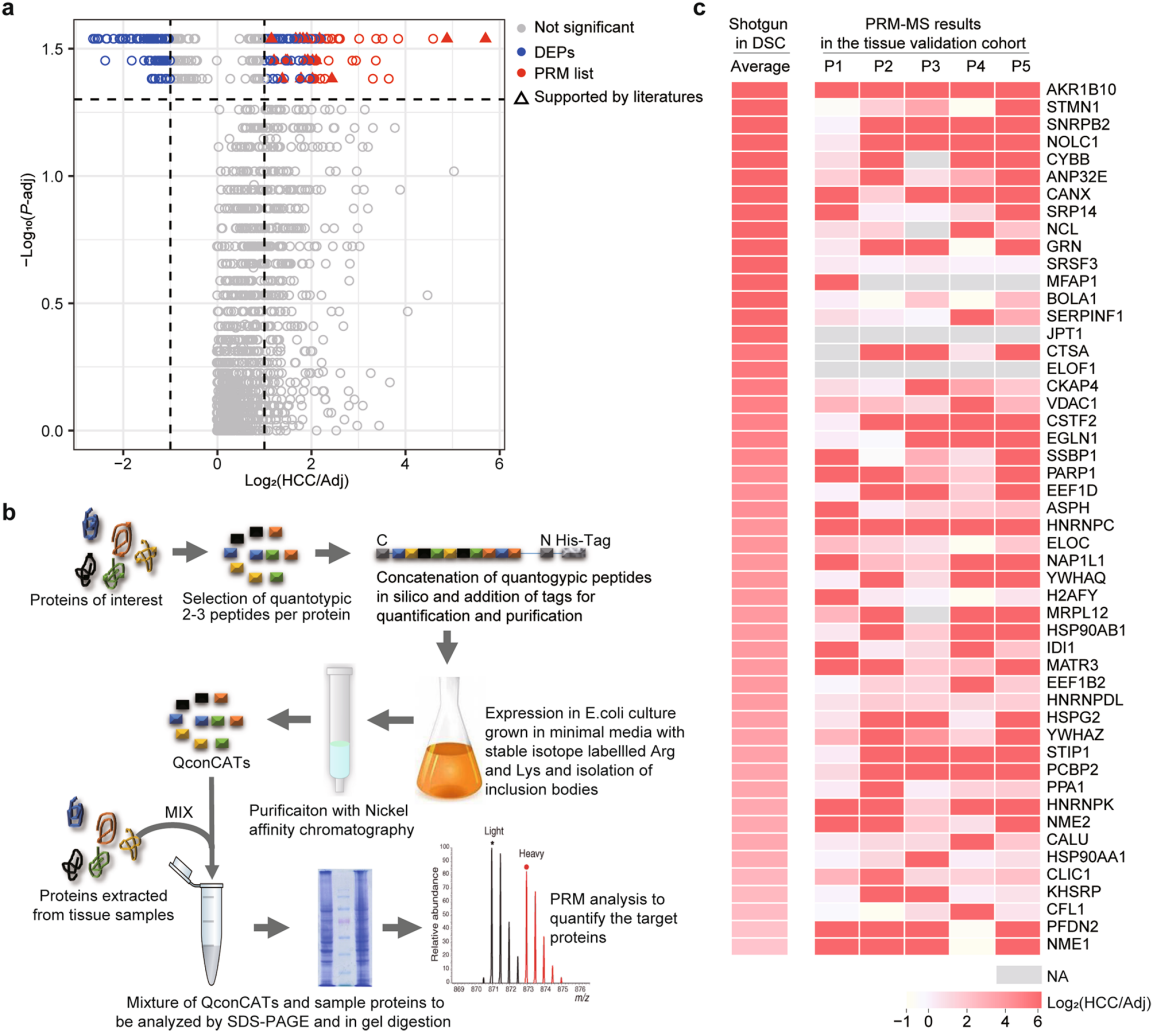
Selection of candidate biomarkers and their validation by PRM-MS assays. **a** The volcano plot of proteomic data with the log_2_-transformed fold-changes (HCC/Adj) as x axis and the − log_10_-transformed adjusted *P* values as y axis. The differentially expressed proteins (DEPs) with log_2_-transformed fold-change ≥ 2 (red circle), or with log_2_-transformed fold-change ≥ 1 and supported by literatures (red triangle) were chosen as potential biomarkers. **b** The scheme of parallel reaction monitor (PRM)-based mass spectrometry (MS). **c** The heatmap of the PRM-MS results in the tissue validation cohort. The color is coded by the log_2_-transformed fold-changes (HCC/Adj). The top ranked 50 potential biomarkers were tested by PRM-MS in 5 paired HCC and adjacent non-tumor liver tissues (P1–P5) of an independent cohort (tissue validation cohort). Left, the average log_2_-transformed fold-changes (HCC/Adj) of the quantitative proteomic results in the discovery cohort (DSC). Adj, adjacent non-tumor liver tissues; QconCAT, quantitative concatemer. NA, not available.

Next, to select potential biomarkers for HCC detection, we focused on the up-regulated proteins in HCC tissues compared to the adjacent non-tumor liver tissues. Therefore, the proteins met one of the following two criteria were selected for validation analyses: (1) proteins with log_2_-transformed fold-change (HCC/Adjacent non-tumor) ≥ 2; (2) proteins with log_2_-transformed fold-change (HCC/Adjacent non-tumor) ≥ 1 and supported by literatures. As a result, a total of 50 up-regulated proteins survived as potential biomarkers for further evaluation (Fig. 3a and Supplementary Table S4).

### Verification of candidate biomarkers in HCC tissue samples by PRM-MS

Next, we sought to elucidate whether the up-regulation of candidate biomarkers in HCC tissues could be reproduced in an independent HCC cohort by an independent method. These 50 potential biomarkers were then analyzed by PRM-MS in paired tumor tissues and adjacent non-tumor liver tissues of 5 HCC patients (designated as the tissue validation cohort; Supplementary methods; Table 1 and Fig. 3b). Among the 50 proteins, 48 ones were reliably detected by PRM-MS; and further, among which a total of 33 proteins were found to be consistently up-regulated (log_2_-transformed fold-change ≥ 1 in at least 4 patients) in HCC tissues compared to the non-tumor liver tissues (Fig. 3c and Supplementary Table S5). Therefore, these 33 proteins might be potential biomarker for HCC detection in tissue samples.

As we aimed to identify serum biomarkers for HCC detection, the proposed potential biomarkers should also have the following features: (1) it should be a secretory or membrane protein that can be released into blood; 2) it can be detected in blood reliably using a convenient and cost-effective method, such as ELISA. Therefore, a total of 8 out of the 33 candidate biomarkers were selected for further evaluation in serum (Supplementary Table S5).

### Clinical validation of the candidate biomarkers in serum samples by ELISA

To ascertain the diagnostic relevance of the 8 candidate biomarkers, we performed ELISA analyses in two independent HCC cohorts of serum samples.

**Figure 4:**
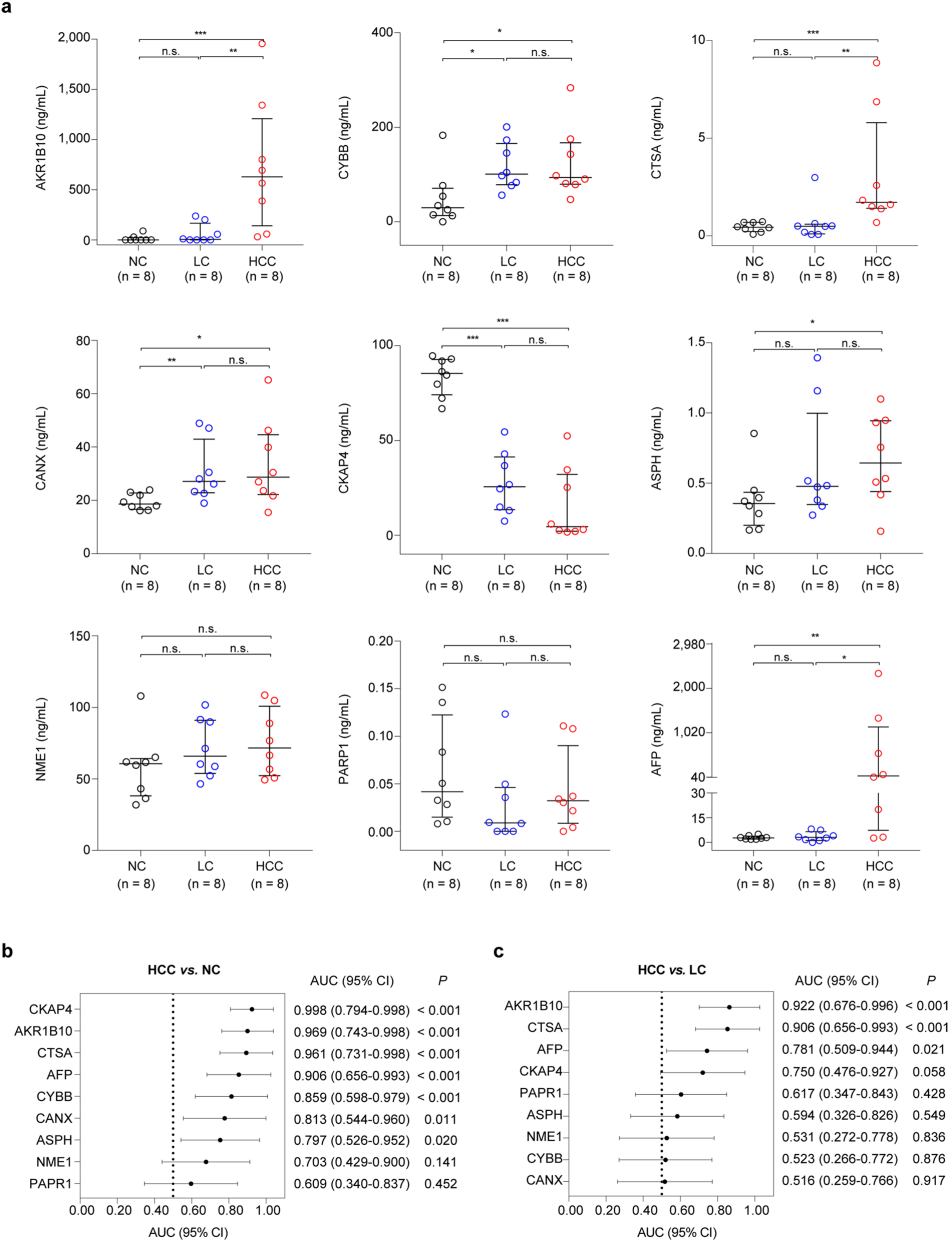
Performance of candidate serum biomarkers and AFP in the serum validation cohort 1. **a** Serum levels of candidate biomarkers and AFP in the serum validation cohort 1, which contains 8 HCC cases (HCCs), 8 liver cirrhosis cases (LCs) and 8 healthy normal controls (NCs). In the plots, each dot represents one sample, with lines representing the median with interquartile range. Mann–Whitney test was used to compare the protein levels between two groups. **b** Performance of the candidate biomarkers discriminating the HCCs from NCs. **c** Performance of the candidate biomarkers discriminating the HCCs from LCs. Bars in (**b**, **c**) indicate the area under the receiver operating characteristic curve (AUC) (95% confidence interval [CI]). *P* values were calculated using the Wilcoxon rank-sum test. AFP, alpha-fetoprotein; AKR1B10, aldo–keto reductase family 1 member B10; ASPH, aspartate beta-hydroxylase; CANX, calnexin; CYBB, cytochrome b-245 beta chain; CKAP4, cytoskeleton associated protein 4; CTSA, cathepsin A; HCC, hepatocellular carcinoma; PARP1, poly (ADP-ribose) polymerase 1; NME1, nucleoside diphosphate kinase A. *P < 0.05, **P < 0.01 and ***P < 0.001. n.s., not significant.

The alteration of protein levels in serum might not be consistent with that in tumor tissues^20^. Thus, we first evaluated individually the performance of the candidate biomarkers, and AFP, which is a currently used diagnostic biomarker for HCC and used as a positive control, in serum of an independent HCC validation cohort (designated as serum validation cohort 1) for initial validation and biomarker selection. This cohort included 8 HCC cases (HCCs), 8 liver cirrhosis cases (LCs), and 8 healthy normal controls (NCs) (Table 1). We observed that the serum levels of aldo–keto reductase family 1 member B10 (AKR1B10), cytochrome b-245 beta chain (CYBB), cathepsin A (CTSA), alnexin (CANX), aspartate beta hydroxylase (ASPH), and AFP were significantly higher in HCCs compared with NCs (*P* < 0.05), while the levels of cytoskeleton associated protein 4 (CKAP4) were significantly lower in HCCs compared with NCs (*P* < 0.05) (Fig. 4a and Supplementary Table S6). Moreover, their areas under the receiver operating characteristic curve (AUCs) were all greater than 0.750 (Fig. 4b). Strikingly, the serum levels of AFP, AKR1B10, and CTSA were also higher in HCCs than in LCs (*P* < 0.05; Fig. 4a and Supplementary Table S6), each with an AUC greater than 0.750 (Fig. 4c), suggesting they might be used to detect HCCs from high-risk populations. Therefore, we chose AKR1B10, CTSA, and AFP for further evaluation.

**Figure 5:**
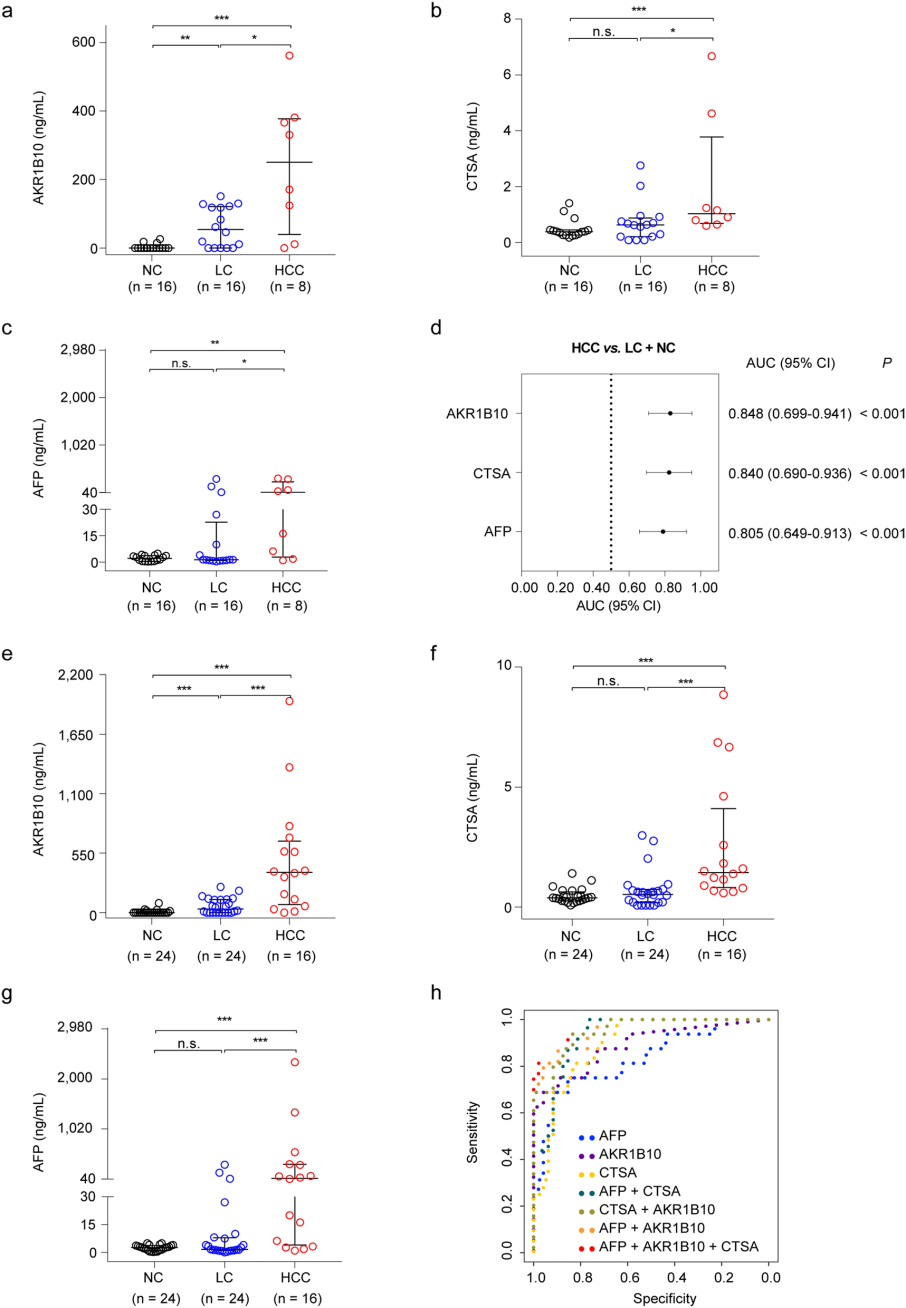
Performance of AKR1B10, CTSA and AFP in the serum validation cohorts. **a–c** Serum levels of AKR1B10, CTSA and AFP in the serum validation cohort 2, which contains 16 HCC cases (HCCs), 16 liver cirrhosis cases (LCs) and 8 healthy normal controls (NCs). **d** Performance of the candidate biomarkers discriminating the HCCs from LCs and NCs in the serum validation cohort 2. Bars indicate the area under the receiver operating characteristic curve (AUC) (95% confidence interval [CI]). *P* values were calculated using the Wilcoxon rank-sum test. **e–g** Serum levels of AKR1B10, CTSA, and AFP in combined samples of the serum validation cohort 1 and 2. **h** Receiver operating characteristics (ROC) curves of the candidate biomarkers discriminating HCCs from LCs and NCs in the samples from the two serum validation cohorts. The multi-protein diagnostic models were constructed by binomial logistic regression analyses. AFP, alpha-fetoprotein; AKR1B10, aldo–keto reductase family 1 member B10; CTSA, cathepsin A. For (**a**–**c**) and (**e**–**g**), each dot represents one sample, with lines representing the median with interquartile range. Mann–Whitney test was used to compare the protein levels between two groups. *P < 0.05, **P < 0.01 and ***P < 0.001. n.s., not significant.

We then subjected AKR1B10, CTSA, and AFP to validation by ELISAs in serum of another independent HCC cohort (designated as serum validation cohort 2; Table 1), which consists of 8 HCCs, 16 LCs, and 16 NCs (Fig. 5a–c). The results confirmed the findings in the serum validation cohort 1. As a positive control, AFP showed higher levels in HCCs [median (min–max): 41.780 [0.938–332.300] ng/mL) than that in LCs (1.350 [0.283–318.100] ng/mL) and NCs (2.050 [0.210–4.970] ng/mL) (all *P* < 0.05, Mann–Whitney test). The serum levels of AKR1B10 in HCCs (250.500 [0.000–562.200] ng/mL) were significantly higher than that in LCs (53.750 [0.000–151.000] ng/mL) and NCs (0.000 [0.000–26.210] ng/mL) (all *P* < 0.05, Mann–Whitney test). Also, the serum concentrations of CTSA in HCC (1.024 [0.592–6.674] ng/mL) were significantly higher than that in LCs (0.625 [0.075–2.758] ng/mL) and NCs (0.380 [0.167–1.404] ng/mL) (*P* < 0.05, Mann–Whitney test). The AUCs for these three proteins distinguishing HCCs from LCs and NCs were all greater than 0.800 (Fig. 5d). Together, these results suggested that AKR1B10 and CTSA might be candidate serum biomarkers for HCC detection.

To comprehensively compare the performance of AKR1B10, CTSA, and AFP in detecting HCC, we merged the two serum validation cohorts. The results showed that the levels of the three proteins were all significantly higher in HCCs than in LCs and NCs (all *P* < 0.05; Fig. 5e–g). Notably, the AUCs for distinguishing HCCs from LCs and NCs were 0.891 and 0.894 for AKR1B10 and CTSA, respectively, compared to the performance of AFP (AUC = 0.831; Fig. 5h). We next explored the potential values of the combination measurements of these proteins as diagnostic models using binomial logistic regression analyses. The results showed that the AFP + AKR1B10 + CTSA model (AUC = 0.969) was superior to the AFP + AKR1B10 model (AUC = 0.958), AKR1B10 + CTSA model (AUC = 0.952) and AFP + CTSA model (AUC = 0.932) (Fig. 5h and Table 2). Of them, the performance of AFP + AKR1B10 + CTSA model and AFP + AKR1B10 model was significantly higher than AFP alone in detecting HCC (*P* < 0.05; Table 2). Collectively, our findings indicated that AKR1B10 and CTSA might be candidate serum biomarkers for HCC detection, and would provide higher performance in combination with AFP.

**Table 2:** Performance of AFP and candidate biomarkers distinguishing HCC cases from liver cirrhosis cases and healthy normal controls in the serum validation cohorts.

Diagnostic models^a^	AUC (95% CI)	*P*^b^	Sensitivity (95% CI)	Specificity (95% CI)	Cutoff^c^	*P*^d^ (vs. AFP)
AFP	0.831 (0.716–0.913)	< 0.01	75.0% (47.60–92.70%)	85.4% (72.2–93.9%)	4.97 ng/mL	–
AKR1B10	0.891 (0.788–0.955)	< 0.01	75.0% (47.6–92.7%)	89.6% (77.3–96.5%)	122.29 ng/mL	0.46
CTSA	0.894 (0.792–0.957)	< 0.01	100.0% (79.4–100.0%)	64.5% (49.5–77.8%)	0.56 ng/mL	0.38
AFP + CTSA	0.932 (0.841–0.980)	< 0.01	100.0% (79.4–100.0%	77.1% (62.7–88.0%)	0.12	0.09
CTSA + AKR1B10	0.952 (0.867–0.990)	< 0.01	93.7% (69.8–99.8%)	83.3% (69.8–92.5%)	0.11	0.08
AFP + AKR1B10	0.958 (0.876–0.992)	< 0.01	87.5% (61.7–98.4%)	89.6% (77.3–96.5%)	0.15	0.035
AFP + AKR1B10 + CTSA	0.969 (0.892–0.996)	< 0.01	93.7% (69.8–99.8%)	85.4% (72.2–93.9%)	0.12	0.029

*AFP* alpha-fetoprotein, *AKR1B10* aldo–keto reductase family 1 member B10, *AUC* areas under the receiver operating characteristic curve, *CI* confidence interval, *CTSA* cathepsin A, *ROC* receiver operating characteristic.

^a^The multi-protein diagnostic models were constructed by binomial logistic regression analyses.

^b^*P* values for the significance of differences between AUCs and 0.5 by Wilcoxon rank-sum test.

^c^The optimal cut-point values in ROC analyses were defined by Youden index.

^d^*P* values for the significance of AUC differences between candidate biomarkers and AFP by Wilcoxon rank-sum test.

### Serum AKR1B10 decreased after surgical removal of primary HCC tumors

**Figure 6:**
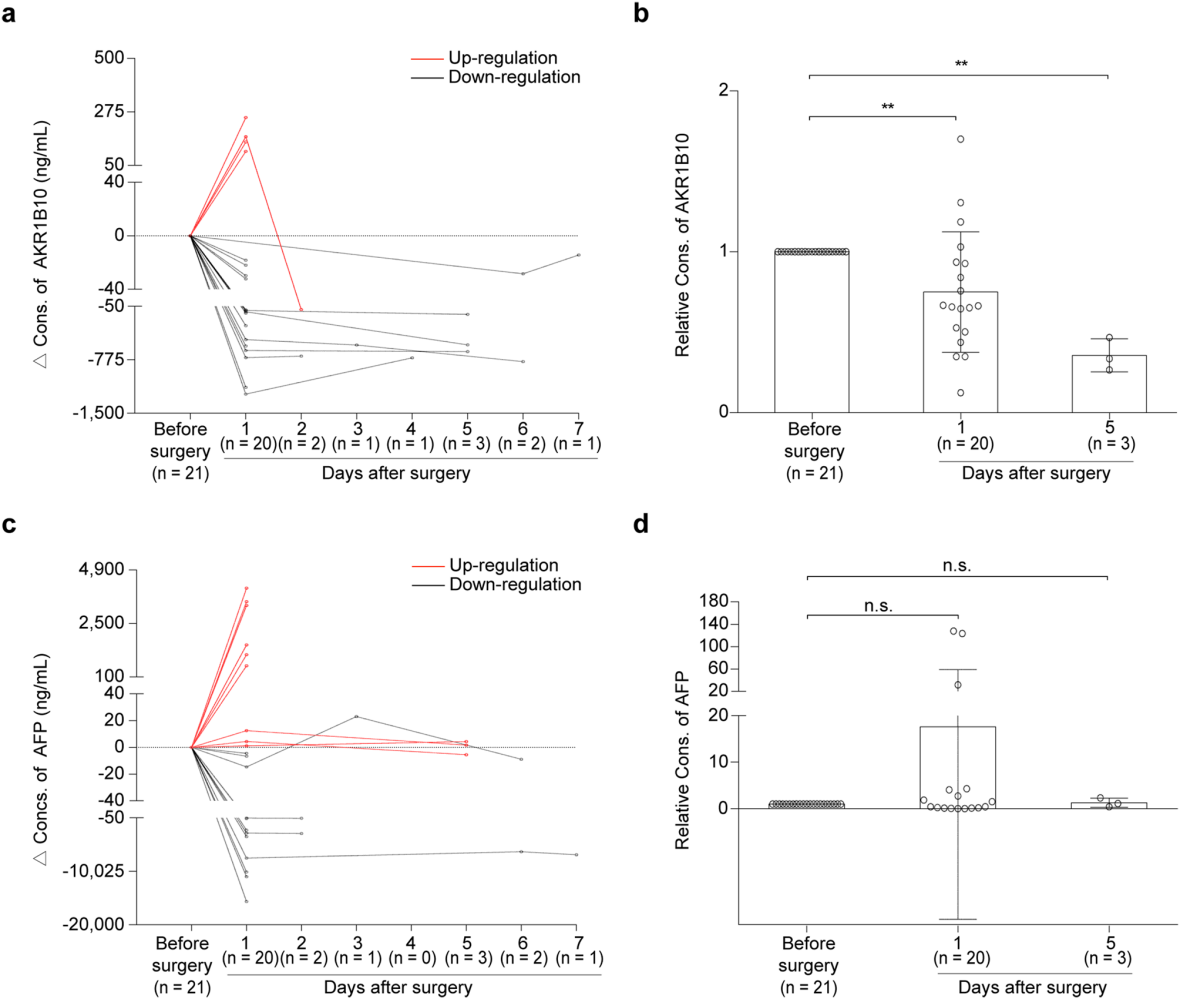
Dynamic changes of serum AKR1B10 and AFP levels after surgical resection of HCC mass. Tendency charts of serum AKR1B10 (**a**) and AFP (**c**) levels in HCC patients before surgery and within one week after operation from the follow-up cohort, which includes 21 HCC patients. For each patient, the protein levels at each day were subtracted by the corresponding baseline concentration (the concentration before surgery). The serum levels of AKR1B10 (**b**) and AFP (**d**) before surgery and at the 1st and the 5th day after surgery in the follow-up cohort. For each patient, the protein levels at each day were divided by the corresponding baseline concentration (the concentration before surgery). Mann–Whitney test was used to compare the protein levels before surgery and after surgery. AFP, alpha-fetoprotein; AKR1B10, aldo–keto reductase family 1 member B10; Cons., concentrations. **P < 0.01. n.s., not significant.

It has been reported that the higher expression levels of AKR1B10 in HCC tumor tissues were significantly associated with larger tumor size and advanced disease stage^27^. Therefore, we further investigated the dynamic changes of serum concentrations of AKR1B10 by ELISA in an independent cohort of 21 HCC patients who were followed up for one week after surgical removal of primary tumors (designated as follow-up cohort; Table 1 and Fig. 6). AFP was measured in parallel for comparison. A total of 51 serum samples were collected from these 21 HCC patients before and within one week after surgical resections (Supplementary Table S7): 21 ones at the day before surgery, 20 at the 1st day, 2 at the 2nd day, 1 at the 3rd day, 1 at the 4th day, 3 at the 5th day, 2 at the 6th day and 1 at the 7th day after surgery. The results showed that, except being not detected in one patient, the serum AKR1B10 levels decreased in 16 of the 21 HCC patients (76.2%), while increased in 4 patients (19.0%) at the 1st day after surgery. Together, the serum levels of AKR1B10 before surgery was 1 163.968 ± 1 810.311 ng/mL, and the values decreased at the 1st day (950.524 ± 1 821.843 ng/mL) after surgery and declined nearly five-fold at the 5th day (228.837 ± 98.622 ng/mL) (*P* < 0.05; Fig. 6b and Supplementary Table S7), suggesting the specificity of serum AKR1B10 concentration for HCC. However, except for the insufficient serum sample before surgery in one patient, the serum AFP levels decreased in only 11 patients (52.4%), while increased in 9 patients (42.9%) at the 1st day after surgery (Fig. 6c). The average serum AFP levels didn’t decline after surgery either at the 1st day or the 5th day (*P* > 0.05; Fig. 6d). Notably, serum AFP levels were less than 20 ng/mL in 6 patients (28.6%) before surgery (Supplementary Table S7), suggesting the limited sensitivity of AFP in the detection of HCC. Taken together, these results highlighted the specificity of serum AKR1B10 for HCC, and also imply its potential usage as a postoperative monitoring biomarker, for which AFP was not competent.

## Discussion

This study aimed to identify valuable serum biomarkers for HCC detection by a 3-stage proteomic study. Dysregulated tissue proteins are presumably the main source of potential blood biomarkers^28^. Therefore, HCC tissues bear great potential in screening HCC biomarker. Several studies have identified a collection of potential diagnostic biomarkers from HCC tissue proteomes, however, few of them have evaluated the clinical value of these potential biomarkers in blood samples^29, 30^. In this study, we first identified dysregulated proteins in HCC tissues using iTRAQ-based proteomic analyses. Then the top-ranked up-regulated proteins were replicated via PRM-MS in an independent tissue cohort. Further, the candidate biomarkers were validated by ELISA in independent multiple cohorts of serum samples. Finally, we found AKR1B10 and CTSA might be potential serum biomarkers for HCC detection, and the combination of AKR1B10, CTSA, and AFP showed a better diagnostic performance than AFP alone.

AKR1B10 is secreted through lysosomes-mediated non-classical secretory pathway. It can detoxify reactive free radical carbonyl compounds and is involved in retinoid metabolism, thus modulates cell proliferation, differentiation and tumorigenesis^31^. AKR1B10 is primarily expressed in the colon and small intestine but over-expressed in several types of cancer, such as non-small cell lung carcinoma and breast cancer^32^. Several studies have reported that AKR1B10 was up-regulated in HCC tissues and might be a potential biomarker for HCC detection^27, 33^, which is in accordance with our results. CTSA is a serine carboxy peptidase, which is involved in catabolic processes and chaperone-mediated autophagy by triggering degradation of lysosome-associated membrane protein type 2a (LAMP2)^34^. CTSA may play important roles in malignant transformation and metastatic dissemination of malignant melanoma^35^. Its roles in HCC, however, have never been illustrated. To our best knowledge, this is the first report of CTSA as a potential diagnostic biomarker for HCC. Notably, the sensitivity of CTSA seems higher than some previously reported potential biomarkers, including AFP-L3 (28–56%)^9^, DCP (56–77%)^8^, and GPC3 (47.9–66.2%)^12^. Further investigations are warranted in the future in independent cohorts.

Interestingly, we also found that there were significant differences between LCs and NCs for serum levels of CKAP4, CYBB, and CANX (all *P* < 0.05; Fig. 4a), each with an AUC greater than 0.800 (Supplementary Fig. S4). These results suggested that these three proteins might be useful in detecting severe liver diseases. It is known that sometimes the alteration of protein levels in serum may not be consistent with that in tumor tissues^20^. Indeed, we observed that the serum CKAP4 levels were down-regulated, but remarkably up-regulated in HCC tissues (Fig. 3c). CKAP4 is a trans-membrane protein and has been identified as a cell surface receptor for the tissue plasminogen activator, surfactant protein A and anti-proliferative factor^36^. Although CKAP4 was reported to be involved in tumor progression and metastasis^37^, its roles in liver diseases have not been reported. CYBB belongs to the enzymes involved in reactive oxygen species (ROS) stress, oxidative injury and wound healing. It has been reported that biliary atresia patients with higher CYBB expression levels had higher risk of LC^38^, suggesting the association between CYBB and LC. CANX is an endoplasmic reticulum-induced protein, and has been reported to be a prognostic marker and potential therapeutic target in colorectal cancer^39^. However, its roles in liver diseases have not been reported. Overall, further studies are needed to evaluate the potential roles of these proteins in liver disease surveillance.

There are a few limitations in our study. First, similar to previous iTRAQ studies^40, 41^, the depth of our iTRAQ-based proteomic analyses (~ 1 983 proteins) was also limited. Second, the number of participants recruited in the validation cohorts remained small and the male–female ratio of HCC patients (> 4:1) differed from the real-world data (2.44:1)^1^, thus, the results should be interpreted with caution and further evaluation for AKR1B10 and CTSA are necessary in larger independent clinical cohorts. In addition, the striking decrease of serum AKR1B10 concentrations after surgery suggests its potential usage as a surveillance biomarker to assess the therapeutic response of HCC patients. However, the changes of serum CTSA levels after surgery were not evaluated due to the limited serum volumes. To further explore the postoperative monitoring roles of these two proteins, we are constructing a long-term and large follow-up cohort.

In conclusion, we, for the first time, combined the high-throughput iTRAQ-based proteome-wide discovery analyses and the medium-throughput PRM-MS validation analyses to systematically identify candidate HCC biomarkers. Besides the highlighted potential biomarkers in tissue samples, we evaluated the 8 top-ranked candidate serum biomarkers via ELISA in serum samples and revealed that serum AKR1B10 and CTSA have potentials for HCC detection. Notably, their combination with AFP yielded a better diagnostic performance, which may improve the detection of HCC from high-risk populations.

## Supplementary information

Supplementary file1

Supplementary file2

## Data Availability

All data that support the findings of this study are included in this published article and its Supplementary information files. The raw files of proteome dataset can be obtained from iProX database (www.iprox.org, Accession No. IPX0002199001).

## References

[1] Bray F (2018). Global cancer statistics 2018: GLOBOCAN estimates of incidence and mortality worldwide for 36 cancers in 185 countries. CA Cancer J. Clin..

[2] Mittal S, El-Serag HB (2013). Epidemiology of hepatocellular carcinoma: consider the population. J. Clin. Gastroenterol..

[3] Villanueva A, Hernandez-Gea V, Llovet JM (2013). Medical therapies for hepatocellular carcinoma: a critical view of the evidence. Nat. Rev. Gastroenterol. Hepatol..

[4] Siegel R, Naishadham D, Jemal A (2013). Cancer statistics, 2013. CA Cancer J. Clin..

[5] Yu NC (2011). CT and MRI improve detection of hepatocellular carcinoma, compared with ultrasound alone, in patients with cirrhosis. Clin. Gastroenterol. Hepatol..

[6] Li D, Satomura S (2015). Biomarkers for hepatocellular carcinoma (HCC): an update. Adv. Exp. Med. Biol..

[7] Gupta S, Bent S, Kohlwes J (2003). Test characteristics of alpha-fetoprotein for detecting hepatocellular carcinoma in patients with hepatitis C. A systematic review and critical analysis. Ann. Intern. Med..

[8] Lok AS (2010). Des-gamma-carboxy prothrombin and alpha-fetoprotein as biomarkers for the early detection of hepatocellular carcinoma. Gastroenterology.

[9] Li D, Mallory T, Satomura S (2001). AFP-L3: a new generation of tumor marker for hepatocellular carcinoma. Clin. Chim. Acta.

[10] Ye Q, Ling S, Zheng S, Xu X (2019). Liquid biopsy in hepatocellular carcinoma: circulating tumor cells and circulating tumor DNA. Mol. Cancer.

[11] Shen Q (2012). Serum DKK1 as a protein biomarker for the diagnosis of hepatocellular carcinoma: a large-scale, multicentre study. Lancet Oncol..

[12] Jia X, Liu J, Gao Y, Huang Y, Du Z (2014). Diagnosis accuracy of serum glypican-3 in patients with hepatocellular carcinoma: a systematic review with meta-analysis. Arch. Med. Res..

[13] Shang S (2012). Identification of osteopontin as a novel marker for early hepatocellular carcinoma. Hepatology.

[14] Tian L (2011). Serological AFP/Golgi protein 73 could be a new diagnostic parameter of hepatic diseases. Int. J. Cancer.

[15] Hu B, Tian X, Sun J, Meng X (2013). Evaluation of individual and combined applications of serum biomarkers for diagnosis of hepatocellular carcinoma: a meta-analysis. Int. J. Mol. Sci..

[16] Ge T (2015). Diagnostic values of alpha-fetoprotein, dickkopf-1, and osteopontin for hepatocellular carcinoma. Med. Oncol..

[17] Keshishian H (2017). Quantitative, multiplexed workflow for deep analysis of human blood plasma and biomarker discovery by mass spectrometry. Nat. Protoc..

[18] Sun W (2016). Quantitative proteomics analysis of tissue interstitial fluid for identification of novel serum candidate diagnostic marker for hepatocellular carcinoma. Sci. Rep..

[19] Xu X (2016). Global proteomic profiling in multistep hepatocarcinogenesis and identification of PARP1 as a novel molecular marker in hepatocellular carcinoma. Oncotarget.

[20] Zhang J (2017). In-depth proteomic analysis of tissue interstitial fluid for hepatocellular carcinoma serum biomarker discovery. Br. J. Cancer.

[21] Kim KH (2019). Parallel reaction monitoring with multiplex immunoprecipitation of N-glycoproteins in human serum for detection of hepatocellular carcinoma. Anal. Bioanal. Chem..

[22] Cui Y, He L, Yang CY, Ye Q (2019). iTRAQ and PRM-based quantitative proteomics in early recurrent spontaneous abortion: biomarkers discovery. Clin. Proteomics.

[23] Babu N (2018). Identification of potential biomarkers of head and neck squamous cell carcinoma using iTRAQ based quantitative proteomic approach. Data Brief..

[24] Xu W, Huang H, Yu L, Cao L (2015). Meta-analysis of gene expression profiles indicates genes in spliceosome pathway are up-regulated in hepatocellular carcinoma (HCC). Med. Oncol..

[25] Jiang Y (2019). Proteomics identifies new therapeutic targets of early-stage hepatocellular carcinoma. Nature.

[26] Gao Q (2019). Integrated proteogenomic characterization of HBV-related hepatocellular carcinoma. Cell.

[27] DiStefano JK, Davis B (2019). Diagnostic and prognostic potential of AKR1B10 in human hepatocellular carcinoma. Cancers (Basel).

[28] Zhu Y (2019). Identification of protein abundance changes in hepatocellular carcinoma tissues using PCT-SWATH. Proteomics Clin. Appl..

[29] Naboulsi W (2016). Quantitative tissue proteomics analysis reveals Versican as potential biomarker for early-stage hepatocellular carcinoma. J. Proteome Res..

[30] Megger DA (2013). Proteomic differences between hepatocellular carcinoma and nontumorous liver tissue investigated by a combined gel-based and label-free quantitative proteomics study. Mol. Cell Proteomics.

[31] Luo DX (2011). Aldo-keto reductase family 1, member B10 is secreted through a lysosome-mediated non-classical pathway. Biochem. J..

[32] Ma J (2012). AKR1B10 overexpression in breast cancer: association with tumor size, lymph node metastasis and patient survival and its potential as a novel serum marker. Int. J. Cancer.

[33] Han C, Gao L, Bai H, Dou X (2018). Identification of a role for serum aldo-keto reductase family 1 member B10 in early detection of hepatocellular carcinoma. Oncol. Lett..

[34] Cuervo AM, Mann L, Bonten EJ, d'Azzo A, Dice JF (2003). Cathepsin A regulates chaperone-mediated autophagy through cleavage of the lysosomal receptor. EMBO J..

[35] Kozlowski L, Wojtukiewicz MZ, Ostrowska H (2000). Cathepsin A activity in primary and metastatic human melanocytic tumors. Arch. Dermatol. Res..

[36] Li SX (2014). CKAP4 inhibited growth and metastasis of hepatocellular carcinoma through regulating EGFR signaling. Tumour. Biol..

[37] Kimura H (2016). CKAP4 is a Dickkopf1 receptor and is involved in tumor progression. J. Clin. Invest..

[38] Wang J (2019). Correlation between hepatic oxidative damage and clinical severity and mitochondrial gene sequencing results in biliary atresia. Hepatol. Res..

[39] Ryan D (2016). Calnexin, an ER stress-induced protein, is a prognostic marker and potential therapeutic target in colorectal cancer. J. Transl. Med..

[40] Zhang Q (2017). Eight-plex iTRAQ labeling and quantitative proteomic analysis for human bladder cancer. Am. J. Cancer Res..

[41] Lin L (2017). Application of iTRAQ-based quantitative proteomics approach to identify deregulated proteins associated with liver toxicity induced by polygonum multiflorum in rats. Cell Physiol. Biochem..

[42] Bruix, J., Sherman, M. & American Association for the Study of Liver, D. Management of hepatocellular carcinoma: an update. *Hepatology* **53**, 1020–1022, 10.1002/hep.24199 (2011). 10.1002/hep.24199 PMC3084991 21374666

[43] Kinoshita A (2015). Staging systems for hepatocellular carcinoma: current status and future perspectives. World J. Hepatol..

