# Supplementary material for: Quantitative proteomics identifies a plasma multi-protein model for detection of hepatocellular carcinoma
Source: Sci Rep. 2020 Sep 23;10:15552. doi: 10.1038/s41598-020-72510-9 (PMC7511324; doi:10.1038/s41598-020-72510-9)
Supplement: Supplementary file 1 — Supplementary file1 [file 41598_2020_72510_MOESM1_ESM.doc]

**SUPPLEMENTARY MATERIALS**

**Quantitative Proteomics Identifies a Plasma Multi-Protein Model for Detection of Hepatocellular Carcinoma**

**Running title:** Candidate Proteins for HCC detection

Zhenhua Du^1*^, Xinyi Liu^1*^, Xiaojun Wei^1^, Hongbo Luo^2^, Peiyao Li^1^, Mengting Shi^1^, Bingqian Guo^1^, Ying Cui^3^, Zhenglin Su^4^, Jifeng Zeng^4^, Anfeng Si^5🖂^, Pengbo Cao^1🖂^, and Gangqiao Zhou^1,2,6🖂^

^1^State Key Laboratory of Proteomics, National Center for Protein Sciences at Beijing, Beijing Proteome Research Center, Beijing Institute of Radiation Medicine, Beijing 100850, P. R. China;

^2^Medical College of Guizhou University, Guiyang city 550025, P. R. China;

^3^Affiliated Cancer Hospital of Guangxi Medical University, Nanning city 530021, P. R. China;

^4^The No.954 Hospital of PLA, Shannan city 856100, P.R. China;

^5^Department of Surgical Oncology, Jinling Hospital, School of Medicine, Nanjing University, Nanjing city 210002, P.R. China;

^6^Collaborative Innovation Center for Personalized Cancer Medicine, Center for Global Health, School of Public Health, Nanjing Medical University, Nanjing city 211166, P.R. China;

^*^These authors contributed equally: Zhenhua Du and Xinyi Liu.

^🖂^**Corresponding authors:**

Dr. Gangqiao Zhou, State Key Laboratory of Proteomics, National Center for Protein Sciences at Beijing, Beijing Proteome Research Center, Beijing Institute of Radiation Medicine, 27 Taiping Road, Beijing 100850, P. R. China. E-mail: zhougq114@126.com; Phone: 86-010-66931201.
**OR**

Dr. Pengbo Cao, State Key Laboratory of Proteomics, National Center for Protein Sciences at Beijing, Beijing Proteome Research Center, Beijing Institute of Radiation Medicine, 27 Taiping Road, Beijing 100850, P. R. China. E-mail: birchcpb@163.com; Phone: 86-010-61777099.

**OR**

Dr. Anfeng Si, Department of Surgical Oncology, Jinling Hospital, School of Medicine, Nanjing University, 305 Zhongshan East Road, Nanjing city 210002, P.R. China. E-mail: saf2008419@163.com; Phone: 86-025-80864733.

**Supplementary Materials:**

Supplementary Methods

Supplementary Tables

Supplementary Figures 1-4

Supplementary reference

**Supplementary Methods**

**Protein extraction and digestion**

Tissue samples were minced and lysed in lysis buffer (8 M Urea, 100 mM Tris Hydrochloride, pH = 8.0) containing protease and phosphatase inhibitors (1:100; #78445, Thermo Scientific, USA), followed by 1 min sonication (3 seconds [s] on and 3 s off, amplitude 25%). Then, the lysate was centrifuged at 14,000 g for 10 min and the supernatant was collected as the whole tissue extracts. The protein concentrations of the supernatant were determined by BCA assay (#23225, Thermo Scientific, USA) following the manufacturer's protocol. Then, the extracts from each sample (100 μg protein) were reduced with 10 mM dithiothreitol (DTT) at 56 °C for 30 min and alkylated with 10 mM iodoacetamide at room temperature in the dark for additional 30 min. Proteins were then precipitated by ice-cold acetone and re-dissolved in 100 μL triethyl ammonium bicarbonate (TEAB, 100 mM). At last, the proteins were typically digested by sequence-grade modified trypsin (1:50; #V528A, Promega, USA) at 37 °C overnight. After trypsin digestion, the peptide was dried by vacuum centrifugation (Speed-Vac, Savant Instruments, USA).

**iTRAQ 8-plex labeling and peptide fractionation**

The iTRAQ 8-plex labelling of the trypsin digested protein samples was done according to the manufacturer’s instructions (#4390811, SCIEX, USA). Briefly, one unit of iTRAQ reagent (defined as the amount of reagent required to label 100 μg of protein) was reconstituted in 70 μL isopropanol. Peptide samples (100 μg) were then labeled with different iTRAQ tags by incubation at room temperature for 2 hours (h). To minimize the individual differences of patients and the analytic variations of different analysis batches, 40 μg protein from each non-tumor sample were pooled together to form a quality control sample (QC). Then, a total of three iTRAQ 8-plex kits were used to label the control sample and 20 tissue samples. The eight iTRAQ tags (113, 114, 115, 116, 117, 118, 119 and 121) in the first kit were used to label seven tumor specimens and one QC; the eight iTRAQ tags in the second kit were used to label the corresponding seven non-tumor specimens and one QC; the last kit was used to label the remaining 3 paired tissue specimens and one QC. For each set of labeled specimens, equal amounts of iTRAQ-labelled peptides were mixed and dried by vacuum centrifugation.

Fractionation of the iTRAQ labeled peptide mixture was performed by cation exchange column (SCX) chromatography using a Shimadzu LC-20AB high-performance liquid chromatography (HPLC) pump system (Shimadzu, Kyoto, Japan). First, each set of the mixed labeled sample was re-suspended in buffer A (25 mM NaH_2_PO_4_ in 25% ACN, pH = 2.7) and loaded onto a 4.6 × 250 mm Ultremex SCX column containing 5 μm particles (#00A-4040-B0, Phenomenex, USA). Then, the peptide mixture was eluted at a flow rate of 1 mL/min with a gradient of buffer A for 20 min, 5-35% buffer B (25 mM NaH_2_PO_4_, 1 M KCl in 25% ACN, pH = 2.7) for 11 min, 35-80% buffer B for 1 min, 80% buffer B for 3 min. Then, the column was equilibrated with buffer A for 10 min for the next injection. Elution was monitored by measuring absorbance at 214 nm, and fractions were collected every 1 min. For each experiment, the collected fractions were combined into 20 fractions and dried in a vacuum concentrator (Speed-Vac, Savant Instruments, USA).

**LC-MS/MS analyses**

Proteome profiles were determined by a liquid chromatography-mass spectrometry/mass spectrometry (LC-MS/MS) platform consisting of a Shimadzu LC-20AD nanoHPLC (Shimadzu, Kyoto, Japan) and a TripleTOF 5600 MS (AB SCIEX, MA, USA). Each fraction was re-suspended in buffer A (2% ACN, 0.1% FA) and loaded to a 2 cm C18 trap column (inner diameter 200 μm; pore size, 120 Å; SunChrom, USA), and then separated on a home-made 10 cm C18 column (inner diameter 75 μm; pore size, 120 Å; SunChrom, USA) at 15 μL/min for 4 min. Subsequently, a 44 min gradient was run at 400 nL/min starting from 2% to 35% B (98% ACN, 0.1% FA), followed by 2 min linear gradient to 80%, then 80% B for 4 min, and finally returning to 2% in 1 min. The MS was operated in the information-dependent mode (IDA) with positive polarity at electrospray voltage of 2.5 kV. Full scan MS was performed with a resolving power of greater than or equal to 30,000 full width at half maximum (FWHM) for TOF MS scans. For IDA, survey scans were acquired in 250 ms with a mass range of 350-2000 m/z and as many as 30 product ion scans were collected if exceeding a threshold of 120 counts per second and with a 2+ to 5+ charge-state. A sweeping collision energy setting of 35 ± 5 eV coupled with iTRAQ adjust rolling collision energy was applied to all precursor ions for collision-induced dissociation.

Protein identification and quantification were performed with the ProteinPilot software (version 4.5; Applied Biosystems, USA). The generated MS data was searched using Mascot (version 2.1; Matrix Science, UK) against a protein sequence database from UniProtKB/Swiss-Prot (Release 2018_05). Enzyme specificity was set to trypsin. Carbamidomethyl cysteine, iTRAQ 8 plex (N-term), and iTRAQ 8 plex (K) were set as a fixed modification; whereas, and oxidized methionine was set as variable modifications. A maximum of one missed cleavage was allowed. Proteins were identified with the unused-ProtScore > 1.3, FDR < 0.01 and at least two unique peptides. Proteins shared by at least 7 pairs of samples were considered for further analysis. Before statistical analysis (Wilcoxon signed-rank test), the protein abundance was standardized by QCs and normalized using quantile normalization. Differentially expressed proteins (DEPs) were defined as proteins with log_2_-transformed fold-change (HCC/adjacent non-tumor) ≥ 1 or ≤ -1, and adjusted *P*-value < 0.05 (Benjamin-Hochberg correction).

**Parallel reaction monitor (PRM) analyses**

For each candidate protein, two or three unique peptides were selected according to the following rules^1^: (1) have a unique m/z value that does not overlap with another peptide in the present analysis; (2) ionizable to be detected; (3) must be reproducibly observed. Therefore, a total of four quantitative concatamer (QconCAT) genes were constructed in Sangon Biotech (Shanghai, China), which covered separately 13, 12, 12 and 13 proteins (Supplementary **Table S3**), and were inserted into the pET-21a plasmid vectors (Novagen, USA). Then the recombinant proteins were expressed in BL21(λDE3) *via* isopropyl-β-D-thiogalactoside (IPTG) induction expression system, which was cultured in minimal media with amino acids [^13^C_6_] arginine and [^13^C_6_] lysine, to elicit stable isotope-labeled standard (SIS) peptides. The “heavy” labeled QconCATs were then purified using 6 × His-Tagged Protein Purification Kit (#CW0894, CWBIO, China) and quantified by BCA assay (#DQ111-01, TransGen Biotech, China).

After proteins extraction, the QconCATs were spiked at a known amount. The samples were then run on a 4%-20% SDS-PAGE. Then, gels were fixed in methanol: acetic acid: water (4:1:5) for 1 h, and stained with Coomassie Brilliant Blue R-250 for 4 h. After target protein bands were cut out (~1 × 2 mm), the protein samples were sequentially reduced with 10 mM DTT, alkylated by 50 mM iodoacetamide, and in-gel digested with trypsin (1:50; #V528A, Promega, USA). The resulting peptide mixture was then extracted from the gel fractions and dried using vacuum centrifugation. The dried peptides were dissolved in buffer A (2% ACN, 0.1% FA) and loaded to a 2 cm C18 trap column (inner diameter 200 μm; pore size, 120Å; SunChrom, USA). The eluate was analyzed using an LTQ Orbitrap Velos (Thermo Fisher, USA) in PRM mode. The results were analyzed using Skyline (version 2.6; AB SCIEX, USA). The averaged intensity ratios between the light and heavy peak pairs (“light” peptides from samples and “heavy” peptides from the QconCATs) from the same protein was used to quantify the target proteins. The up-regulated proteins in HCC tissues were defined as the proteins with a log_2_-transformed fold-change ≥ 1 between the paired HCC tissues and adjacent non-tumor liver tissues in ≥ 4 patients.

**Enzyme-linked immunosorbent assay (ELISA)**

The ELISA experiments were performed according to the manufacture’s protocol. Briefly, assay diluent, recombinant protein standards and diluted serum samples were added to appropriate number of wells sequentially. The resulting plate was incubated, and followed by washings. Then, a biotinylated detector antibody was added to each well and incubated. After washing the wells to remove the unbound detector antibody, an avidin-horseradish peroxidase (HRP) conjugate was added and incubated. After washing, an enzymatic reaction was produced through the addition of 3,3',5,5'-tetramethylbenzidine (TMB) substrate which is catalyzed by HRP generating a blue color product that changes to yellow after adding acidic stop solution. The signal was read immediately at 450 nm, using a Tecan’s Sunrise microplate reader (Tecan, SunriseTM, Switzerland).

The assays for aldo-keto reductase family 1 member B10 (AKR1B10), and cytochrome b-245 beta chain (CYBB) were developed in-house:

***The ELISA for AKR1B10*:** First, appropriate number of wells in the ELISA plate (Thermo Scientific™ Immulon with MaxiSorp surface, Thermo Fisher Scientific, Denmark) were coated with 100 μL per well of the mouse antibodies (10 μg/mL; H00057016-M03, Novus Biologicals Inc, USA) and incubated overnight at 4 °C. Then, the wells in the ELISA plate was filled with 200 μL of 5% skimmed milk containing 0.05% Tween-20 per well and kept for 2 hours (h) at room temperature, followed by four washings (PBS containing 0.05% Tween-20). Then, 100 μL of serum samples, assay dilutions, and serial dilutions of recombinant protein were added to the corresponding wells, and the plate was incubated for 2 h at 37 °C, followed by four washings. Next, 100 μL of rabbit anti-human AKR1B10 polyclonal antibody (1:600; 18252-1-AP, Proteintech, USA) were added per well. After 2 h at 37 °C and subsequent four washings, 100 μL of HRP conjugated anti-rabbit IgG polyclonal antibody (1:20,000; #0102-14, Scicrest, China) was added per well and incubated for 1 h at 37 °C. After four washings, 90 μL of TMB substrate (#34580, Thermo Fisher Scientific, Denmark) was added per well and incubated for 15 min at room temperature. At last, the reaction was stopped by adding 50 μL of stop solution (2 M H_2_SO_4_) per well, and then the plate was read immediately at 450 nm using a Tecan’s Sunrise microplate reader (Tecan, SunriseTM, Switzerland).

***The ELISA for CYBB:*** First, appropriate number of wells (Thermo Scientific™ Immulon with MaxiSorp surface, Thermo Fisher Scientific, Denmark) were coated with 100 μL of the mouse antibodies (5 μg/mL; NBP1-41012, Novus Biologicals Inc, USA) and incubated overnight at 4 °C. Then, 200 μL of 5% skimmed milk containing 0.05% Tween-20 were added to each well and kept for 2 h at room temperature, followed by four washings (PBS containing 0.05% Tween-20). Then, 100 μL of serum samples, assay dilutions, and serial dilutions of recombinant protein were added to corresponding wells. After incubation for 2 h at 37 °C and subsequent four washings, 100 μL of rabbit anti-human CYBB polyclonal antibody (1:600; 19013-1-AP, Proteintech, USA) were added per well. After incubation for 2 h at 37 °C and four washings, each well was added with 100 μL of HRP conjugated anti-rabbit IgG polyclonal antibody (1:20,000; #0102-14, Scicrest, China). After 1 h incubation at 37 °C and subsequent four washings, 90 μL of TMB substrate (#34580, Thermo Fisher Scientific, Denmark) was added per well and incubated for 15 min at room temperature. Finally, 50 μL of stop solution (2 M H_2_SO_4_) was added per well to stop the reaction and the signal was read immediately at 450 nm using a Tecan’s Sunrise microplate reader (Tecan, SunriseTM, Switzerland).

**Supplementary Tables**

**Table S1.** Eligibility criteria for the recruited participants.

**Table S2.** Specific unique peptides of the 50 potential protein biomarkers for validation by PRM-MS assays.

**Table S3**. Major demographic and clinicopathological parameters of the 100 participants in this study and the serum levels of candidate biomarkers in these participants.

**Table S4.** The 183 differentially expressed proteins identified in the discovery cohort by iTRAQ-based proteomic analyses.

**Table S5.** The PRM-MS results of the 50 potential biomarkers in the tissue validation cohort.

**Table S6.** Summary of the serum levels of the 8 candidate biomarkers and AFP in the serum validation cohort 1.

**Table S7.** The serum levels of AKR1B10 and AFP in the follow-up cohort.

**Supplementary Figures**

**Figure. S1.** The distribution and correlation of quality controls (QCs).

**Figure. S2.** The KEGG pathways enriched by the differentially expressed proteins.

**Figure. S3.** Expression heatmap of the differentially expressed proteins enriched in the spliceosome pathway.

**Figure. S4.** Performance of the candidate biomarkers discriminating the liver cirrhosis cases from healthy normal controls in the serum validation cohort 1.


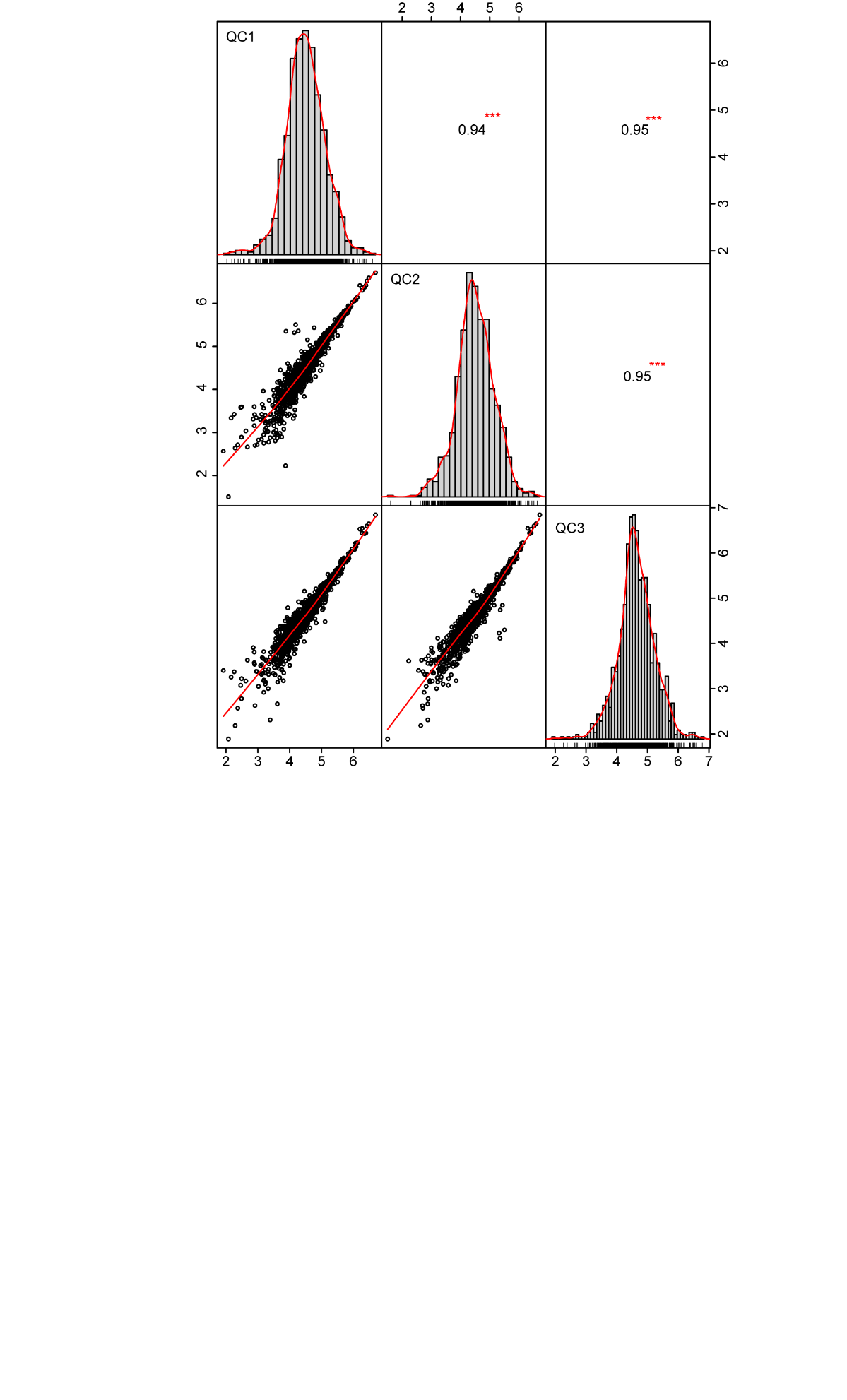


**Figure. S1.** The distribution and correlation of quality controls (QCs). The distribution of log_2_-transformed intensity of the identified proteins in each QC that were prepared by mixing aliquots of each non-tumor sample is shown on the diagonal. The Pairwise scatterplots of the three QCs are shown on the bottom of the diagonal, and the corresponding Pearson correlation coefficients are shown on the top of the diagonal. ^***^*P* < 0.001.


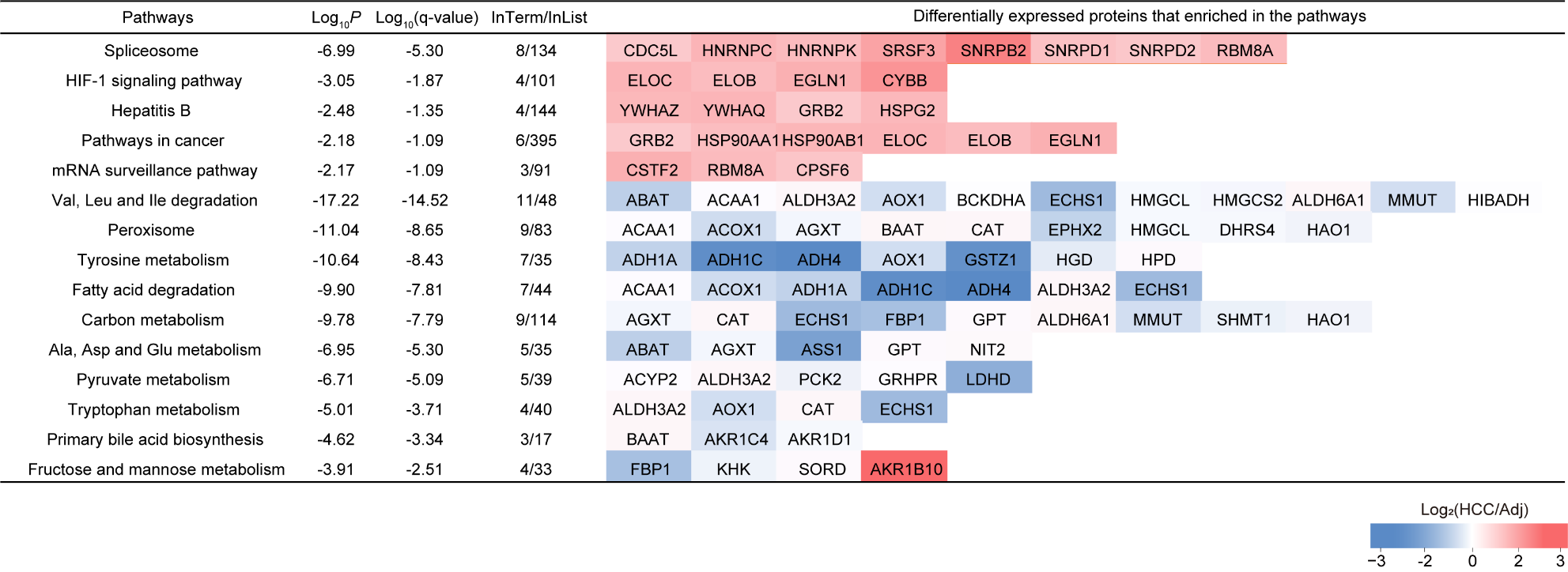


**Figure. S2.** The KEGG pathways enriched by the differentially expressed proteins. The Kyoto Encyclopedia of Genes and Genomes (KEGG) pathway enrichment analyses were performed using Metascape. The color is coded by the average log_2_-transformed fold-changes (HCC/Adj) of the quantitative proteomic results in the discovery cohort (DSC). Adj, adjacent non-tumor liver tissues. Ala, alanine; Asp, aspartate; Glu, glutamate; Ile, isoleucine; InTerm/InList, how many genes are enriched – InTerm, versus how many total genes are in a given functional category – InList; Leu, leucine; Val, valine.


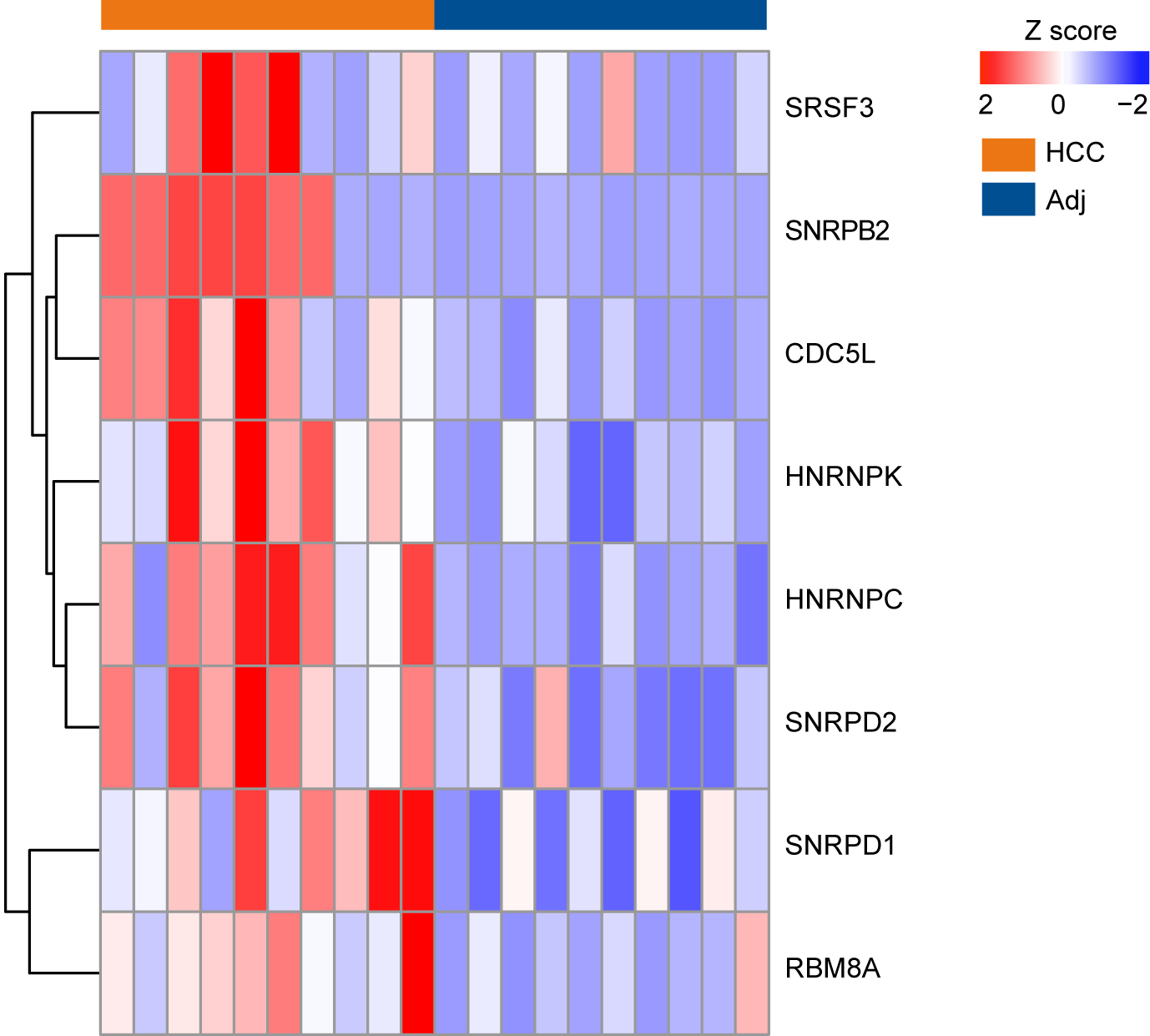


**Figure. S3.** Expression heatmap of the differentially expressed proteins enriched in the spliceosome pathway. Adj, adjacent non-tumor liver tissues; CDC5L, cell division cycle 5 like protein; HCC, hepatocellular carcinoma; HNRNPC, heterogeneous nuclear ribonucleoprotein C; HNRNPK, heterogeneous nuclear ribonucleoprotein K; RBM8A, RNA binding motif protein 8A; SRSF3, serine and arginine rich splicing factor 3; SNRPB2, small nuclear ribonucleoprotein polypeptide B2; SNRPD1, small nuclear ribonucleoprotein D1 polypeptide; SNRPD2, small nuclear ribonucleoprotein D2 polypeptide.


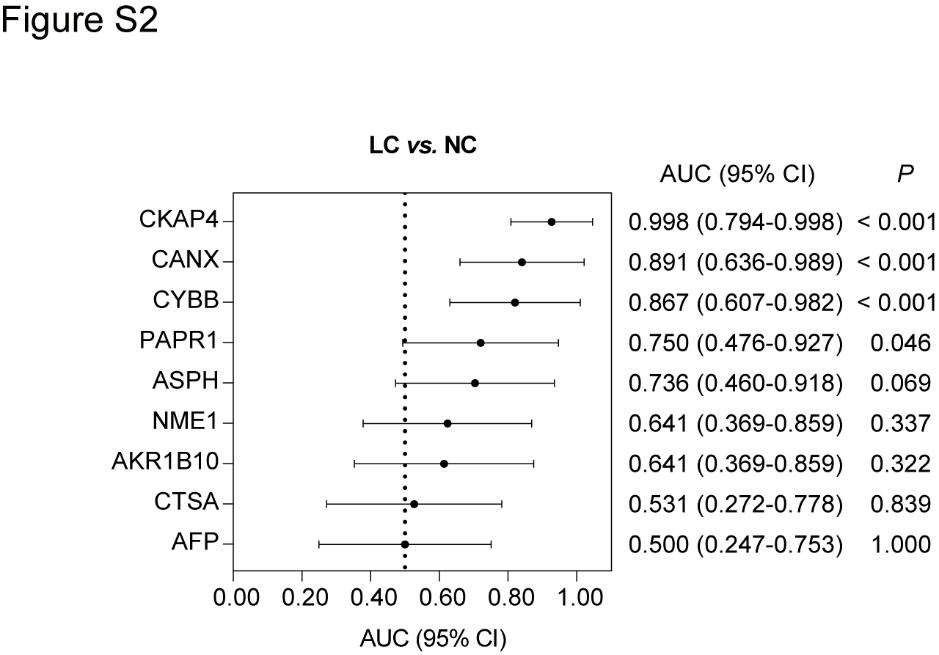


**Figure. S4.** Performance of the candidate biomarkers discriminating the liver cirrhosis cases from healthy normal controls in the serum validation cohort 1. Bars indicate the area under the receiver operating characteristic curve (AUC) (95% confidence interval [CI]). *P* values were calculated using the Wilcoxon rank-sum test. AFP, alpha-fetoprotein; AKR1B10, aldo-keto reductase family 1 member B10; ASPH, aspartate beta-hydroxylase; CANX, calnexin; CYBB, cytochrome b-245 beta chain; CKAP4, cytoskeleton associated protein 4; CTSA, cathepsin A; LC, liver cirrhosis; PARP1, poly (ADP-ribose) polymerase 1; NC, healthy normal controls; NME1, nucleoside diphosphate kinase A.

**References**

1 Brownridge, *P. et a*l. Global absolute quantification of a proteome: Challenges in the deployment of a QconCAT strategy*. Proteomi*c**s** 11, 2957-2970, doi:10.1002/pmic.201100039 (2011).
